# Tissue distribution and transcriptional regulation of CCN5 in the heart after myocardial infarction

**DOI:** 10.1007/s12079-021-00659-7

**Published:** 2021-12-01

**Authors:** Sima Zolfaghari, Ole Jørgen Kaasbøll, M. Shakil Ahmed, Fabian A. Line, Else Marie V. Hagelin, Vivi T. Monsen, Håvard Attramadal

**Affiliations:** 1 Institute for Surgical Research, Oslo University Hospital, Rm. A3.1056, Sognsvannsveien 20, Nydalen, P.O. Box 4950, 0424 Oslo, Norway; 2 Institute of Clinical Medicine, University of Oslo, Oslo, Norway

**Keywords:** β_2_-Adrenergic receptor, Catecholamines, CCN5, CCN2, Myocardial infarction, Primary cardiac fibroblasts

## Abstract

CCN5 is a divergent member of the cellular communication network factor (CCN) family in that it lacks the carboxyl terminal cystine knot domain common to the other CCN family members. CCN5 has been reported to antagonize the profibrotic actions of CCN2 and to inhibit myocardial collagen deposition and fibrosis in chronic pressure overload of the heart. However, what mechanisms that regulate CCN5 activity in the heart remain unknown. Recombinant, replication defective adenovirus encoding firefly luciferase under control of the human CCN5 promoter was prepared and used to investigate what mechanisms regulate CCN5 transcription in relevant cells. Tissue distribution of CCN5 in hearts from healthy mice and from mice subjected to myocardial infarction was investigated. Contrary to the profibrotic immediate early gene CCN2, we find that CCN5 is induced in the late proliferation and maturation phases of scar healing. CCN5 was identified principally in endothelial cells, fibroblasts, smooth muscle cells, and macrophages. Our data show that CCN5 gene transcription and protein levels are induced by catecholamines via β_2_-adrenergic receptors. Myocardial induction of CCN5 was further confirmed in isoproterenol-infused mice. We also find that CCN5 transcription is repressed by TNF-α, an inflammatory mediator highly elevated in early phases of wound healing following myocardial infarction. In conclusion, CCN5 predominates in endothelial cells, fibroblasts, and macrophages of the differentiating scar tissue and its transcription is conversely regulated by β_2_-adrenergic agonists and TNF-α.

**Supplementary Information:**

The online version contains supplementary material available at 10.1007/s12079-021-00659-7.

## Introduction

Ischemic heart disease, also known as coronary artery disease, is one of the leading causes of human morbidity and mortality (Moran et al. 2014). Following acute thrombosis of a coronary artery, ischemia of the myocardial region supplied by the artery ensues (Ambrose and Singh 2015). Unless immediate revascularization of the occluded artery is accomplished, ischemic necrosis of myocardial tissue occurs (Ambrose and Singh 2015). Based on the limited capacity of myocardial tissue to regenerate, tissue lost in ischemic necrosis is replaced by scar tissue by mechanisms similar to general wound healing (Eming et al. 2014). Release of chemoattractants from activated platelets and necrotic cells causes recruitment of inflammatory leucocytes (neutrophils and macrophages) that clear the injured area from dead cells and debris (Frangogiannis 2008). This inflammatory phase is subsequently followed by a proliferative phase with formation of granulation tissue that ultimately differentiates into a mature scar tissue (Ren et al. 2002; Frangogiannis 2008). Previous research has emphasized on ischemia–reperfusion injury and on unravelling novel mechanisms of cardioprotection in order to reduce myocardial necrosis (Altamirano et al. 2015). However, these efforts have yet not translated to new therapies. Thus, current treatment of acute myocardial infarction focuses on rapid revascularization of the occluded artery in order to rescue myocardial tissue from irreversible damage (Ibanez et al. 2018). Still, several pathophysiological mechanisms that operate during scar healing may ultimately have profound impact on ventricular remodeling and cardiac function, e.g. infarct area dilatation, removal of necrotic tissue by granulocytes and macrophages, neutrophil efferocytosis, progression of myofibroblasts into senescence and a senescence associated phenotype that keep on releasing a number of proinflammatory molecules (Prabhu and Frangogiannis 2016). Interestingly, matricellular CCN proteins have been reported to be involved in several of these mechanisms. CCN proteins are secreted proteins that play important roles in several aspects of wound healing, i.e. inflammation, angiogenesis and fibrosis (Jun and Lau 2011). The CCN protein family comprises 6 members typically characterized by having four conserved homology domains, i.e. an insulin-like growth factor-binding protein homology domain (IGFBP), a von Willebrand factor type C homology domain (VWC), a thrombospondin type 1 repeat (TSP) homology domain, and a C-terminal domain (CT) with a cystine knot motif (Bork 1993; Brigstock 1999). CCN5, formerly known as WNT1 inducible signaling pathway protein 2 (WISP-2), is a divergent member of the family in that it lacks the carboxyl-terminal cystine knot domain (Zhang et al. 1998). Indeed, CCN5 has been reported to have many opposite functions of CCN2 (Connective Tissue Growth Factor, CTGF). Whereas CCN2 have been implicated in myocardial fibrosis in heart failure of both ischemic and non-ischemic etiologies, mice with cardiac-restricted overexpression of CCN5 have been reported to exhibit attenuated myocardial fibrosis following chronic pressure overload of the heart, indicating an antagonistic role of CCN5 relative to that of CCN2 in the heart (Yoon et al. 2010). In this respect, previous studies revealed an inhibitory effect of CCN5 on proliferation and motility in smooth muscle cells (Lake et al. 2003; Mason et al. 2004) and fibroblasts (Xu et al. 2015), i.e. the opposite of the functions often ascribed to CCN2. In addition, CCN5 has been reported to inhibit transforming growth factor-β (TGF-β)-induced transdifferentiation of fibroblasts to myofibroblasts by diminishing expression of profibrotic mediators (Xu et al. 2015). While the distribution and regulation of CCN2 in the heart following myocardial infarction is well described, (Chen et al. 2000; Ahmed et al. 2004; Chuva de Sousa Lopes et al. 2004) that of CCN5 is largely unknown.

The aims of this study were to (1) investigate the cellular distribution and regulation of CCN5 in the heart following myocardial infarction (MI) and (2) elucidate the transcriptional regulation of CCN5 in cardiac fibroblasts and endothelial cells.

## Materials and methods

### Cell culture

Human embryonic kidney 293 cells (HEK293A) (R70507, Thermo Fisher Scientific, Waltham, USA), human embryonic lung fibroblasts (IMR-90) (ATCC CCL-186, LGC Standards, Germany) and primary murine cardiac fibroblasts (isolated from mouse hearts) were maintained in Dulbecco’s modified Eagle’s medium (DMEM) with high glucose (Gibco, Cat # 41965-062, USA), supplemented with 10% fetal bovine serum (FBS) (Gibco, Cat # 16000-044, USA) and 50 µg/ml gentamycin sulphate (Sanofi, Cat # 453130, Norway). Human Umbilical Vein Endothelial Cells (HUVEC) (ATCC CRL-1730, LGC Standards, Germany) were maintained in Endothelial cell Basal Medium (EBM) (Lonza, Cat # CC-3121, Switzerland), including the EGM SingleQuots growth supplements (Lonza, Cat # CC-4133, Switzerland) and 50 µg/ml gentamycin sulphate.

### Plasmids

For generation of recombinant adenovirus encoding luciferase-reporter under control of the CCN5 promoter, the pacAd5 shuttle vector (Anderson et al. 2000) was first engineered to incorporate a Gateway™ destination vector cassette (pGL4.10 from Promega, Madison, USA) upstream of the coding sequence of firefly luciferase. The human CCN5 promoter (− 1800/+ 21) was custom-synthesized (ThermoFisherScientific) with flanking attB Gateway™-recombination sequences, and through standard Gateway™ ligation-independent cloning, transferred to the pacAd5 shuttle vector (Fig. SI-a). Truncated variants of the promoter (− 1191/+ 21, − 884/+ 21, and − 389/+ 21) were generated by classical restriction enzyme subcloning techniques. A deletion of the putative cAMP response element (CRE): tgacctca (Fritah et al. 2006) of the − 398/+ 21 CCN5 promoter-reporter construct was custom-synthesized and also transferred to the adenoviral shuttle vector by Gateway-cloning. All synthesized constructs were verified by DNA sequencing analysis.

### Adenovirus preparations

Infectious, replication-defective recombinant adenoviruses were generated in HEK293A cells following co-transfection of recombinant shuttle vector and the RAPAd viral backbone vector using Lipofectamin™ LTX Reagent with Plus™ Reagent (Invitrogen, Cat# 15338100, USA). Recombinant viruses were subsequently amplified and purified with CaptoCore700 HiTrap™ (GE17-5481-51, GE Healthcare, Boston, USA) size-exclusion/anion exchange chromatography columns as described previously (Kaasboll et al. 2018), and titered with the Adeno-X rapid Titer kit from Clontech (Cat# 631028, Takara Bio, USA) according to manufacturer’s protocol. Adenoviruses were aliquoted and stored at − 80 °C until further use.

### CCN5 promoter-reporter assay

IMR-90 cells, primary cardiac fibroblasts and HUVEC cells were seeded at densities of 10,000 cells/well (96-well plates), 25,000 cells/well (24-well plates) and 15,000 cells/well (96-well plates) respectively, transduced with adenovirus encoding luciferase reporter under control of indicated fragment of CCN5 promoter at multiplicity of infection (MOI) of 1000. The cells were incubated overnight in full growth medium, serum-starved for 20–24 h, and subsequently stimulated with indicated concentrations of dexamethasone (Cat# D1756, Sigma-Aldrich, USA), isoproterenol (Norges Apotekerforening, Oslo, Norway), norepinephrine (Norges Apotekerforening, Oslo, Norway), 8-Bromo-cAMP sodium salt (Cat# 1140, TOCRIS, UK), recombinant rat TNF-α protein (510-RT/CF, R&D SYSTEMS, USA), timolol maleate (PHR2593, Sigma-Aldrich), ICI-118,551 hydrochloride (I127, Sigma-Aldrich), atenolol (A7655, Sigma-Aldrich), prazosin hydrochloride (P7791, Sigma-Aldrich), doxazosin mesylate (D9815, Sigma-Aldrich), TPCA-1 (Cat# 2559, TOCRIS, UK) or vehicle for 24 h. At the end of stimulation period, the medium was decanted and 100 µL of ONE-Glo™ substrate / lysis reagent (Cat# E6110, Promega), diluted 1:4 in H_2_O, and was added to each well. The plates were subsequently incubated for 10 min before the lysates were transferred to black-walled plates and luciferase activity was determined by recording of luminescence signals with PolarStar Omega plate reader (BMG LABTECH, Germany).

### Isolation of RNA and quantitative real-time PCR (q-RT-PCR)

Total RNA was isolated from myocardial tissue samples or cells by the RNeasy mini-kit (Cat# 74004, QIAGEN, Hilden, Germany) according to the manufacturer’s instructions. RNA was subsequently reverse-transcribed with the TaqMan Reverse Transcription Reagents Kit (Thermo Fisher Scientific) according to manufacturer’s protocol, and the cDNA generated was utilized for real-time qPCR analyses using TaqMan™ Gene Expression Assays, Fast Advanced Master Mix and the StepOnePlus Real-Time PCR cycler (Thermo Fisher Scientific). mRNA levels are expressed as relative to 18S rRNA. The TaqMan probes used are as follow: Human WISP2 (Assay ID: Hs01031984_m1), Human 18S (Assay ID: Hs99999901_S1), Mouse WISP2 (Assay ID: Mm00497471_m1), Mouse Rn18s (Assay ID: Mm03928990_g1).

### Immunofluorescence staining of IMR-90 cells and myocardial tissue sections

IMR-90 cells were seeded in 24-well plates at a density of 10,000 cells/well on cover glass inserts. Following serum-starvation in 0.1% FBS overnight, the cells were stimulated with 100 nM dexamethasone, 200 nM isoproterenol, 1 mM 8-Bromo-cAMP, 100 nM ICI-118,551, or vehicle for 72 h. Afterwards, the cells were fixed and permeabilized with 4% paraformaldehyde and 0.1% Triton-X100 in phosphate-buffered saline (PBS), respectively. 1% BSA in PBS was used to block non-specific staining. Plates were then incubated overnight at 4 °C with anti-CCN5 antibody (abcam; Cat # ab38317, Cambridge, UK) and subsequently with Alexa Fluor 594-conjugated goat anti-rabbit IgG (H + L) (highly cross-adsorbed) as secondary antibody (Invitrogen; Cat # A-11037) for 1 h at 37 °C before nuclear staining with Hoechst 33258 (Thermo Fisher Scientific). Cover glasses were subsequently placed and fixed on glass slides and a Zeiss Axio Observer Inverted Microscope and ZEN 3.1 (ZEN lite, blue edition) software were used for image analyses.

For double immunofluorescence staining of myocardial tissue, sections were blocked with PBS containing 5% serum and subsequently incubated overnight at 4 °C with primary antibodies against CCN5 (abcam; Cat # ab38317), and CD31 (ER-MP12; Thermo Fisher Scientific) or CD68 (ab201844; Abcam, Inc.). For co-staining of immunoreactive CCN5 and CD31, the sections were subsequently incubated with Alexa 594-labeled goat anti-rabbit IgG and Alexa 488-labelled goat anti-rat IgG (Thermo Fisher scientific). For co-staining of CCN5 and CD68, the sections were incubated with anti-CCN5 IgG (abcam; Cat # ab38317), and anti-CD68 IgG-conjugated with Alexa 488, and subsequently incubated with secondary Alexa 594-labeled goat anti-rabbit IgG. Hoechst 33258 dye solution was used for nuclear staining.

### Western blot analysis of IMR-90 cells

IMR-90 cells were seeded at a density of 9 × 10^5^ cells/100 mm tissue culture dish in DMEM (Gibco, Cat # 41965-062, USA) supplemented with 10% fetal bovine serum (FBS) and 50 µg/ml gentamycin sulphate and maintained in cell incubator for 24 h before medium change and serum-starvation in 0.1% FBS overnight. The cells were subsequently stimulated with 200 nM isoproterenol, 100 nM dexamethasone, or vehicle for 72 h. Cell lysates were prepared in lysis buffer (1% SDS in 10 mM Tris-HCl, pH 8.8), sonicated, and centrifuged at 14,000 × g for 25 min. The protein concentrations of the lysates were determined by the Pierce BCA assay kit (Thermo Fisher Scientific). For each sample, the same amount of protein was loaded and separated by SDS gel electrophoresis using 4–15% TGX gradient gels (Bio-Rad) followed by transfer to a PVDF membrane using the Trans-Blot Turbo semidry blotting system (Bio-Rad). The membrane was blocked in 5% nonfat dry milk dissolved in Tris-buffered saline with Tween 20 (20 mM Tris-HCl, pH 7.4, 140 mM NaCl, 2.5 mM KCl, and 0.1% Tween 20; all chemicals were analytical grade from Merck) for 1 h before probing with primary antibody in 5% nonfat dry milk dissolved in Tris-buffered saline containing 0.1% Tween 20 overnight at 4 °C. The membrane was incubated successively with antibodies against CCN5 (LSBio; cat # LS-C349158) and β-Actin (Cell Signaling; cat # 4970S). Secondary antibody incubation was performed at room temperature for 1 h with HRP-linked anti-rabbit IgG (Cell Signaling; cat # 7074). The membrane was finally incubated with SuperSignal West Femto Maximum Sensitivity Substrate (Thermo Fisher Scientific) and chemiluminescence signals were analyzed with the ChemiDoc imaging system (Bio-Rad). Densitometry of the immunoreactive bands was performed using the Image Lab 6.0.1 software.

### Animals

All animal experiments were performed in accordance with the National Institutes of Health (NIH) Guide for the Care and Use of Laboratory Animals (8th edition, 2011) and were approved by the national board for laboratory animal research, the Norwegian Animal Research Authority (authorization no. 6288).

### Isolation of primary cardiac fibroblasts

12–16 weeks old C57BL/6 female mice were anesthetized with gas anesthesia. Briefly, anesthesia was induced in an induction chamber providing ambient air with 3% isoflurane. During the subsequent procedures anesthesia was maintained by a face mask providing ambient air containing 1% isoflurane. Mice were subsequently euthanized by cervical dislocation and the hearts were rapidly excised, cannulated and perfused with collagenase type 2 (Worthington Biochemical Corp., NJ, USA), as described by O’Connell et al. (2007). Isolated fibroblasts were maintained in 10 cm culture dishes and allowed to adhere for 2 h before removal of unattached cells. The culture media was changed every second day until cell confluence. All experiments were carried out at passage 1–2.

### Experimentally-induced myocardial infarction (MI) and heart failure in mice

Male and female rodents respond similarly with myocardial hypertrophy and left ventricular remodeling following experimental myocardial infarction although the magnitude of the changes may vary (Pfeffer et al. 1979; Wu et al. 2003; Cavasin et al. 2004). However, female mice are considerably less prone to myocardial rupture and thus, the potential bias caused by loss of animals in the experimental groups (Wu et al. 2003). Hence, in this study 12–16 weeks old female C57BL/6 mice were subjected to sham-operation (n = 3) or myocardial infarction (MI) (n = 12) by ligation of the left anterior descending coronary artery (LAD) as described previously by Gao et al. (2010). Before the surgical procedure, anesthesia was induced by gas anesthesia (3% isoflurane). Anesthesia was subsequently maintained by face mask providing a gas mixture of 2–3% isoflurane and 97–98% oxygen. Appropriate depth of anesthesia was controlled by absence of paw reflex. Buprenorphin (0.1 mg/kg s.c.) was provided as analgesia immediately before and after the surgical procedure. The mice subjected to myocardial infarction were randomized to 4 groups (n = 3 in each group) to be euthanized 2, 7, 28, or 50 days after ligation of LAD. For analysis of myocardial mRNA, animals were euthanized 2, 7, 28, or 50 days after induction of myocardial infarction. At the indicated end-points, the mice were anesthetized by gas anesthesia (3% isoflurane) and subsequently euthanized by excision of the heart. Tissue samples from the infarcted part of the left ventricle and the non-ischemic myocardial region were harvested for extraction of RNA (days 2, 7 and 50) or IHC-analyses (28 days).

### Immunohistochemistry of myocardial tissue sections

Myocardial tissue samples from MI- and sham-operated C57BL/6 mice were fixed in 4% paraformaldehyde in phosphate-buffered saline for 1 h, embedded in paraffin wax, and stored at 4 °C. IHC analysis of myocardial sections (6 µm) was performed as previously described (Ahmed et al. 2011), using rabbit polyclonal anti-mouse CCN5 (bs-5100R; Bioss Antibodies Inc., Woburn, USA) and rabbit monoclonal anti-mouse α-smooth muscle actin (α-SMA) (ab124964; Abcam, Inc.). The avidin–biotin–peroxidase system (PK-6100, Vectastain Elite kit; Vector Laboratories, Inc., California, USA) was used for signal amplification. Omission of primary antibody was used as negative controls.

### Continuous infusion of isoproterenol in mice

Myocardial samples from mice subjected to continuous infusion of isoproterenol were used from a previously reported study (Gravning et al. 2013). Briefly, 6 months old male C57BL/6 mice were randomized to continuous subcutaneous infusion of isoproterenol bitartrate (50 mg/kg per day; Sigma-Aldrich) or vehicle for 14 days (vehicle, *n* = 9 and isoproterenol infusion, n = 9). Physical data from echocardiographic examination of these study groups have previously been reported (Gravning et al. 2013). Total RNA from myocardial biopsies from vehicle-infused (n = 6) and isoproterenol-infused (n = 5) mice were isolated and used for analysis of mRNA levels.

### Statistical analysis

Data are shown as the mean ± SEM (n ≥ 3 independent experiments). Statistical analyses were performed using GraphPad Prism, version 6.0. Statistical analysis of differences between two groups was assessed by unpaired Student’s two tailed t-test. Statistical analysis of differences among several experimental groups was performed by one-way ANOVA, followed by Dunnett’s or Šidák’s post hoc test as indicated in the figure legends when relevant. *p* < 0.05 was considered to indicate statistically significant differences.

## Results

### Diverging regulation of CCN2 and CCN5 mRNA after induction of myocardial infarction

**Fig. 1:**
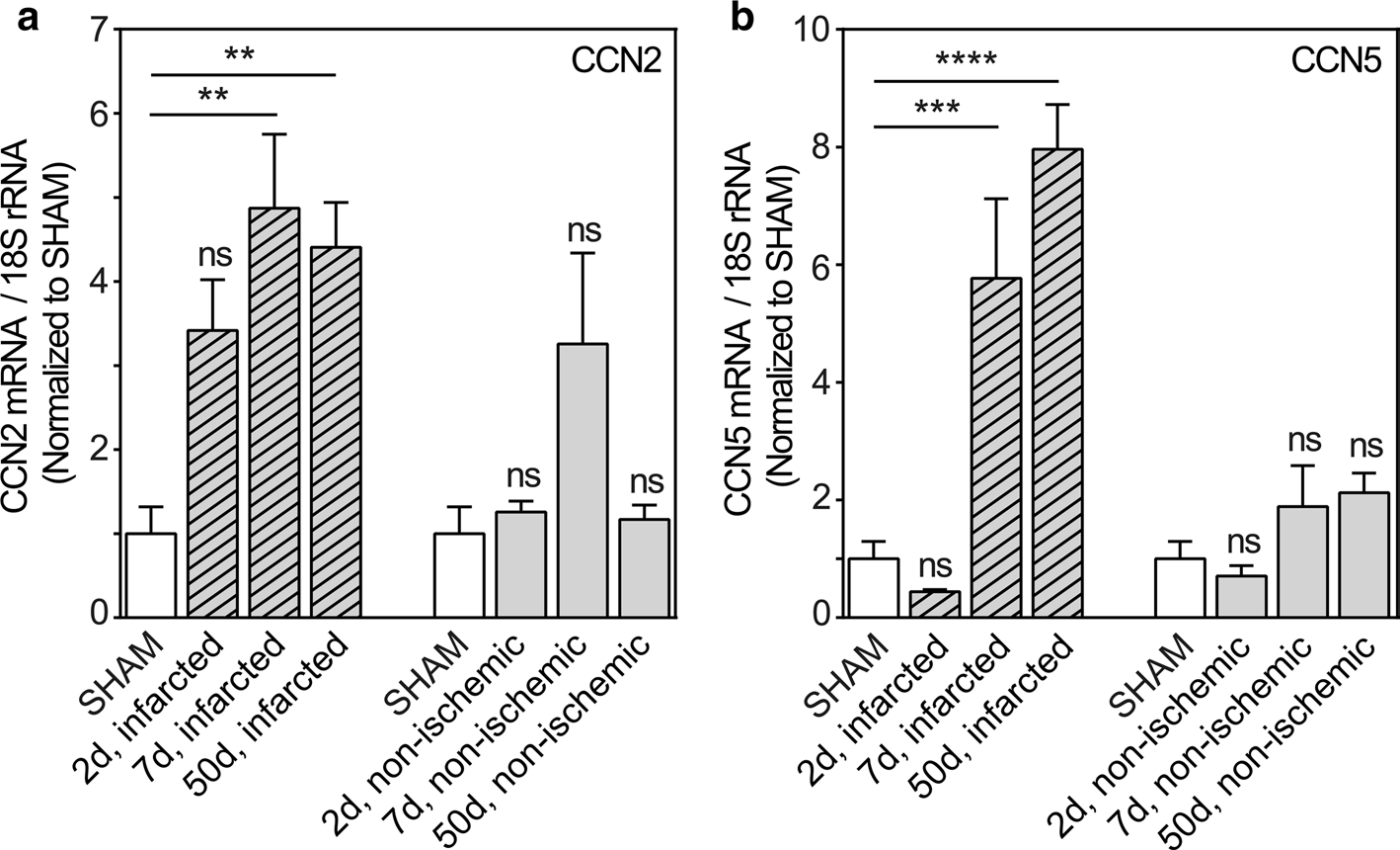
Time course of CCN2 and CCN5 mRNA levels in infarcted and non-ischemic myocardial tissue after induction of experimental myocardial infarction in mice. Histogram demonstrating CCN2 (panel **a**) and CCN5 (panel **b**) mRNA levels relative to 18S rRNA (house-keeping) in infarcted region and in non-ischemic myocardial tissue, harvested 2, 7, and 50 days after permanent ligation of the left coronary artery and normalized to levels in myocardial tissue of sham-operated mice. A column (open column) representing levels in myocardial tissue of sham-operated mice has been included both in front of columns showing levels in the infarcted region and in front of columns demonstrating levels in non-ischemic myocardial tissue. mRNA levels were analyzed by real-time RT-PCR. Data are shown as the mean ± SEM (n = 3 in each group). The data were subjected to statistical analyses by one-way ANOVA with Dunnett’s post hoc test. Statistically significant alterations were indicated by **p < 0.01; ***p < 0.001; ****p < 0.0001 versus sham-operated mice (SHAM); ns: indicates not statistically significant versus sham-operated mice (SHAM)

To decipher the regulatory patterns of CCN2 and CCN5 in tissue repair after myocardial infarction, the mRNA levels of CCN2 and CCN5 in the infarcted region and in non-ischemic myocardial tissue were analyzed at three different time points after induction of myocardial infarction in mice (Fig. 1a, b). CCN2 mRNA levels in the infarcted region increased rapidly following onset of ischemia and remained high during all the phases of wound healing, i.e. the inflammatory phase (day 2 post-MI), the proliferative phase (day 7 post-MI; granulation tissue) and maturation phase (day 50 post-MI; differentiated scar tissue) (Fig. 1a). In contrast to the CCN2 mRNA levels, the increase of CCN5 mRNA levels in the infarcted region was delayed and could first be detected during the proliferative phase of wound healing assayed at day 7 post-MI. CCN5 mRNA levels continued to be elevated in the differentiated scar (up to 50 days post-MI) (Fig. 1b). However, whereas CCN2 mRNA levels appear to display a transient increase in non-ischemic myocardial tissue consistent with previous findings (Ahmed et al. 2004), neither the alterations of CCN2 mRNA levels nor those of CCN5 in non-ischemic myocardial tissue were statistically significant compared with levels in sham-operated mice (Fig. 1a, b).

### Cellular distribution of CCN5 in the heart following myocardial infarction

**Fig. 2:**
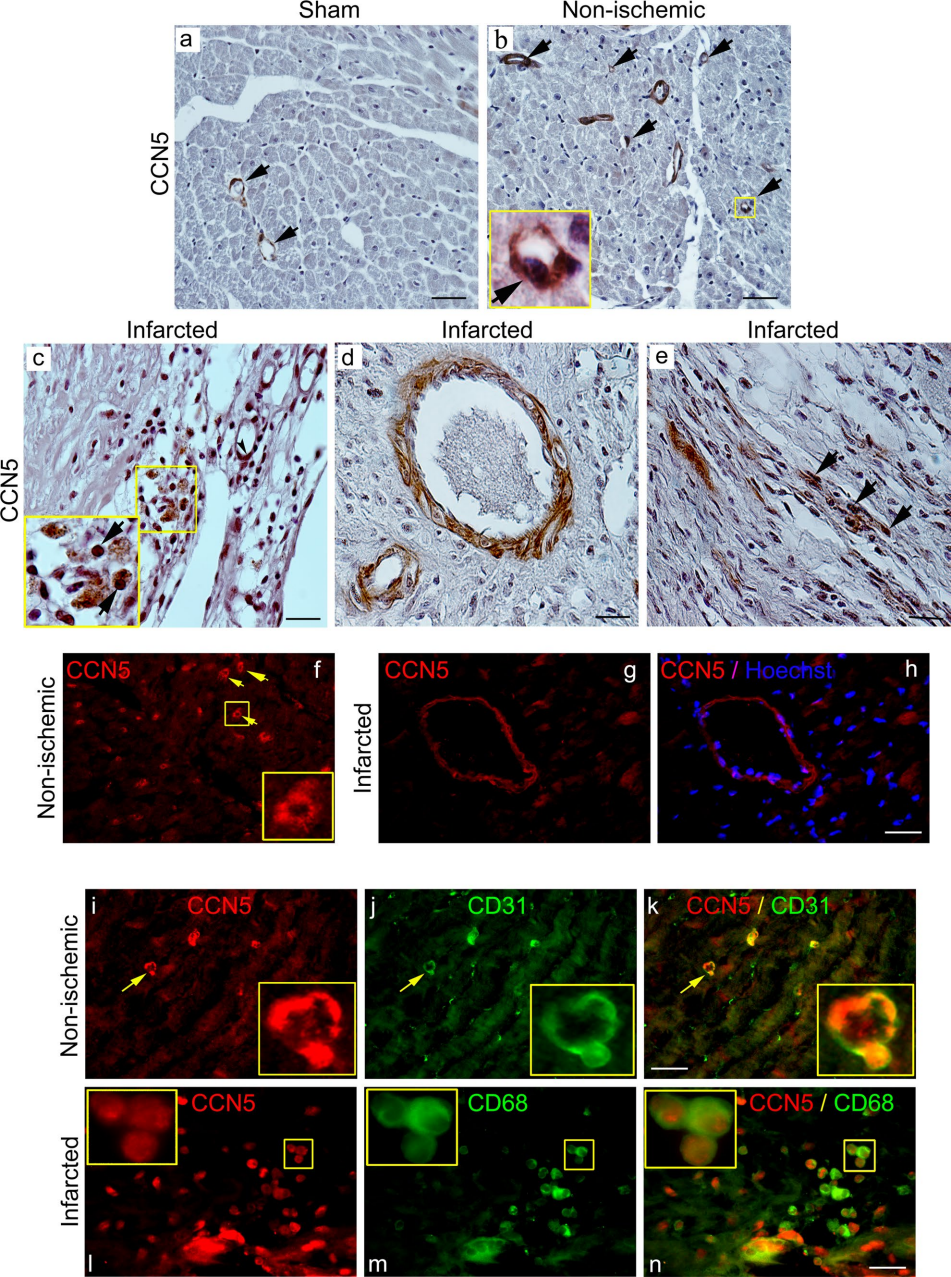
Immunohistochemical and immunocytochemical analysis of CCN5 immunoreactivity in myocardial tissue. The upper panels (**a**–**e**) are photomicrographs of immunohistochemistry of CCN5 immunoreactivity in myocardial tissue sections from **a** sham-operated mice, **b** non-ischemic region or **c**–**e** infarct region of mice 4 weeks after permanent ligation of the left coronary artery and induction of myocardial infarction. The arrows in panels **a** and **b** indicate CCN5 immunoreactivity in endothelial cells of capillaries and small vessels. Panel **c** demonstrates CCN5 immunoreactivity in the differentiating scar tissue 4 weeks after myocardial infarction. The arrows point to immunostaining of mononuclear leukocytes. Panel **d** demonstrates immunostaining of muscular layer of a small artery. Panel **e** demonstrates CCN5 immunoreactivity in elongated, spindle-shaped cells likely to represent fibroblasts (arrowheads, panel **e**). Scale bar is 50 µM for **a** and **b**, and 20 µM for **c**–**e**. Panels **f**–**h** are photomicrographs of immunofluorescence analysis of CCN5 immunoreactivity in non-ischemic myocardial tissue or infarct region following ligation of the left coronary artery in mice. Arrowheads in panel **f** indicate immunostaining of capillaries in non-ischemic myocardial tissue (inlet demonstrates enlargement of immunoreactive capillary). Panel **g** and **h** demonstrate CCN5-positive artery in the infarct region with immunostaining of muscular layer (panel **h** shows overlay with Hoechst nuclear stain). Panels **i**–**k** demonstrate double-staining of CCN5 and CD31 immunoreactivities in non-ischemic myocardial tissue. Panel **k** is an overlay of panels **i** and **j**. Inlets demonstrate 100-fold magnification of immunostaining of capillary indicated by arrowhead and confirming CCN5 immunoreactivity in CD31-positive endothelial cells. Panels **l**–**n** demonstrate double-staining of CCN5 and CD68 reactivities in developing scar tissue 4 weeks after induction of myocardial infarction. Inlet (100-fold magnification in upper left corner of panels) demonstrates CCN5 immunoreactivity in CD68-positive macrophages (overlay in panel **n**). Scale bar is 20 µM for **f**–**n**. Photomicrographs of all panels are representative immunostaining of myocardial sections from 2 sham-operated mice or 2 mice subjected to myocardial infarction (three myocardial sections were subjected to each indicated immunostaining)

The finding of increased CCN5 mRNA levels in the healing wound following myocardial infarction indicates that the cells of the developing scar, such as endothelial cells, fibroblasts, or leukocytes may be the sources of CCN5. To further resolve this issue, tissue sections of hearts sampled from sham-operated mice, as well as from mice 4 weeks after induction of myocardial infarction, was used for immunohistochemical (IHC) and immunofluorescence (IF) staining. Immunohistochemical analysis of the distribution of CCN5 immunoreactivity in tissue sections from sham-operated animals revealed that CCN5 was largely confined to endothelial cells of capillaries and other microvessels (Fig. 2a). Similar distribution of CCN5 was observed in non-ischemic myocardial tissue of the mice subjected to myocardial infarction (Fig. 2b). In the developing scar tissue replacing the myocardial necrosis 4 weeks after induction of myocardial infarction, CCN5 immunoreactivity was observed in endothelial cells of capillaries and other microvessels, mononuclear leukocytes, and spindle-shaped fibroblast-like cells (Fig. 2c–e). CCN5 immunoreactivity was also observed in the muscular layer (smooth muscle cells of tunica media) of larger vessels (Fig. 2d) similar to the distribution of α-smooth muscle actin (α-SMA) immunoreactivity (Fig. SI-b).

In order to determine the cellular origin of CCN5 in the tissue, IF double-staining of cardiac tissue sections from hearts sampled 4 weeks after induction of MI was performed with antibodies against CCN5 and either CD31 (marker of endothelial cells), or CD68 (marker of macrophages). First, immunofluorescence staining of CCN5 revealed that CCN5 immunoreactivity was prevailing in capillaries and other microvessels of both non-ischemic myocardial tissue and of the developing scar (Fig. 2f–h). Co-staining of CCN5 and CD31 immunoreactivities in the myocardial sections confirmed that endothelial cells were the prevailing cell type expressing CCN5 (Fig. 2i–k). Furthermore, cellular co-staining of CCN5 and CD68 also revealed CCN5 expression in macrophages of the developing scar tissue (Fig. 2l–n).

### Identification of human CCN5 promoter region

**Fig. 3:**
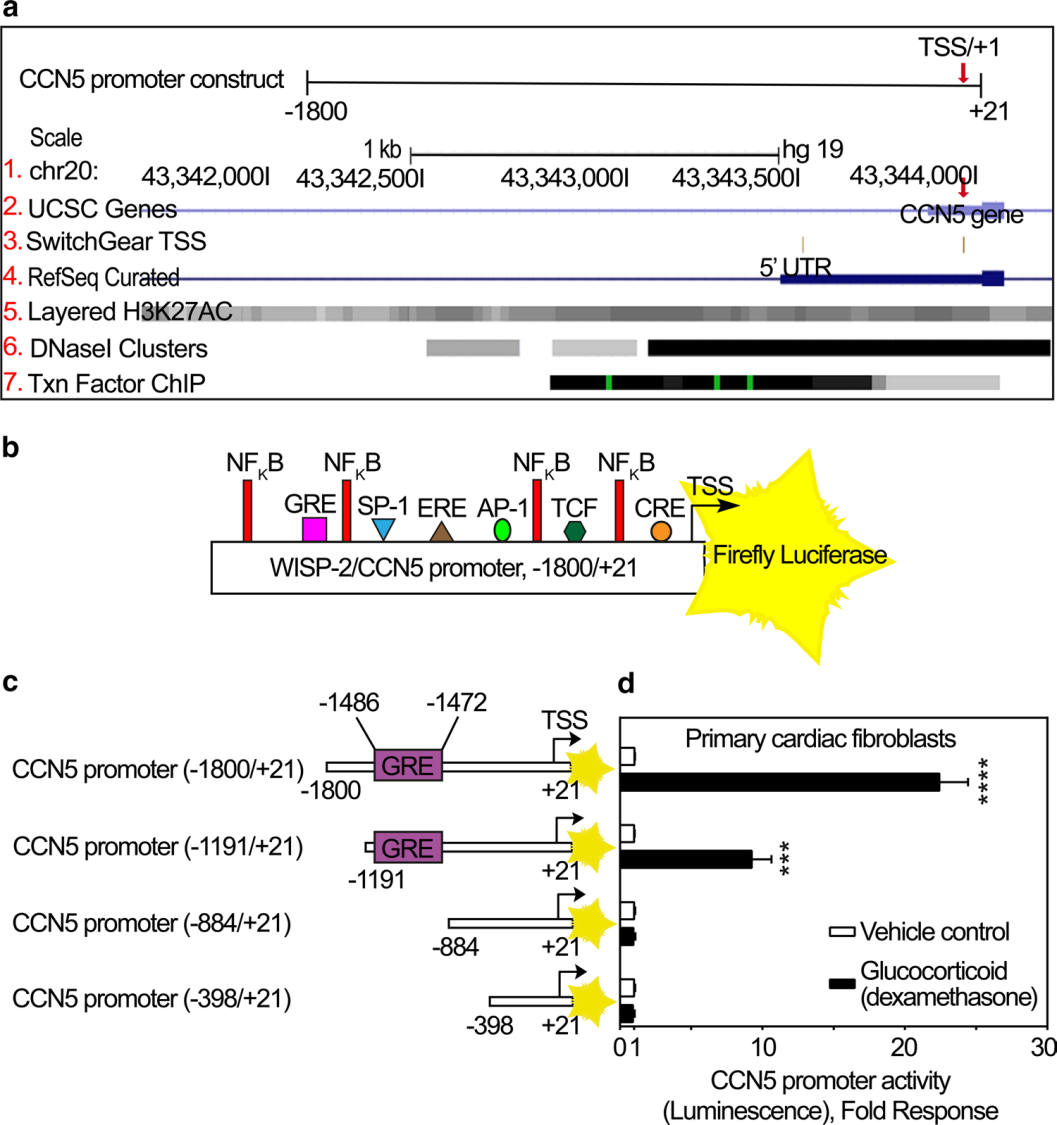
Bioinformatic analysis and deletion studies of the promoter region of CCN5. **a** Schematic illustration of CCN5 promoter region (− 1800/+ 21) upstream of the coding state of CCN5 gene. All tracks were set and displayed as dense. Red arrows demonstrate Transcription Start Site (TSS)/+ 1. (1) CCN5 gene is located on chromosome 20. The numbers are indicative of the base position with the genomic coordinates of the displayed region (gene promoter). (2) Homo sapiens WNT1 inducible signaling pathway protein 2 (WISP-2)/CCN5, mRNA, assembled by UCSC genome browser. (3) SwitchGear TSS: location of Transcription Start Sites (TSSs) throughout the human genome. Red arrow illustrates the chosen TSS/+ 1 with higher confidence score based on reported experimental evidence. (4) Transcript variants alignments of CCN5 according to RNA reference sequences (RefSeq) gene predictions from NCBI. (5) H3K27Ac mark: this involves acetylation of lysine 27 of the H3 histone protein. H3K27Ac is defined as an active enhancer mark often found near active regulatory elements. Overlayed H3K27Ac tracks show where modification of histone proteins is suggestive of enhancer and, to a lesser extent, other regulatory activity. (6) DNase I hypersensitive clusters (from ENCODE analysis) which are accessible chromatin zones, transcriptionally active and necessary for the binding of transcription factors. A gray box designates the degree of the hypersensitive region. The darkness is relative to the maximum signal strength observed in any of the reported 125 cell types. (7) Txn Factor ChIP: transcription factor (161 factors) binding motifs from a large collection of ChIP-seq experiments (chromatin immunoprecipitation followed by sequencing) performed by the ENCODE Factorbook repository. Clusters are showing occupancy regions for each factor and motif sites within the regions. A gray box surrounds each peak cluster of transcription factor occupancy. The darkness is indicative of the maximum signal strength observed in any cell line contributing to the transcription factor occupancy. A green highlight, within a cluster indicates the highest scoring site of a Factorbook-identified established motif for the corresponding transcription factor. **b** Full length WISP-2/CCN5 promoter (− 1800/+ 21) immediately preceding the transcription start site (TSS/+ 1) and coding sequence of firefly luciferase (FLUC) as reporter of CCN5 promoter activity. Consensus enhancer/suppressor elements in the promoter region of CCN5 are predicted and indicated according to the Transfac database. **c** Schematic demonstrating various deletions of the − 1800/+ 21 promoter construct with indications of the previously identified glucocorticoid response (enhancer) element (GRE). After infection of primary cardiac fibroblasts **d** with recombinant adenovirus encoding luciferase under control of the CCN5 promoter fragment with various deletions, the cells were stimulated with 100 nM dexamethasone or vehicle for 24 h and assayed for luciferase activity. The results are presented as the mean ± SEM (n = 3 independent experiments with two replicates per condition) of luciferase activity in primary cardiac fibroblasts stimulated in the absence or presence of dexamethasone. Statistical significance was assessed by unpaired Student’s two tailed t-test. ***p < 0.001 and ****p < 0.0001

To analyze the human CCN5 promoter region, the UCSC (University of California, Santa Cruz) Human Genome Browser Assembly (GRCh37/hg19, Feb. 2009) was used to locate the CCN5 gene within a chromosomal context. The promoter was identified as extending approximately 1500–1600 bp upstream of the transcription start site based on the presence of epigenetic modifications of histone 3 (H3K27AC modifications), DNase I hypersensitivity clusters, (often found near active regulatory elements), and the clustering of ChIP-seq identified transcription factor binding elements (Fig. 3a). To ensure inclusion of all major regulatory elements, we used a fragment of 5′ untranslated CCN5 DNA up to 1800 bp upstream of the transcription start site, thus yielding a construct of − 1800/+ 21 bp to be inserted in-frame with the reporter gene (Fig. 3a).

The Transfac database (TRANSFAC 2016) was hence used to predict potential transcription factor binding sites within the promoter region of CCN5. Figure 3b shows a schematic of the − 1800/+ 21 CCN5 promoter-reporter construct with indication of putative transcription factor binding sites.

### The CCN5 promoter is responsive to glucocorticoids in IMR-90 cells, primary cardiac fibroblasts and HUVEC cells

The promoter region of the CCN5 gene has previously been reported to contain a putative glucocorticoid response (enhancer) element (GRE) (5′-GGTACGTACTGTTCC-3′) and to respond to glucocorticoid stimulation (dexamethasone) in estrogen receptor alpha (ERα)-negative MDA-MB-231 breast cancer cells (Ferrand et al. 2012). The reported GRE in the promoter region of CCN5 is not a consensus GRE as originally proposed (Scheidereit et al. 1983). However, considerable degeneracy of the proposed consensus sequence has later been reported (Del Monaco et al. 1997). In order to confirm the involvement of the reported GRE of the CCN5 promoter to glucocorticoid stimulation, primary cardiac fibroblasts (cFBs) and human lung fibroblasts (IMR-90 cells) were transduced with recombinant adenovirus containing varying lengths of the CCN5 promoter (− 1800/+ 21, − 1191/+ 21, − 884/+ 21 and − 398/+ 21) set to control the firefly luciferase (FLUC) reporter gene (Fig. 3c), and subsequently stimulated in the absence or presence of dexamethasone (100 nM). Consistent with the report on CCN5 promoter responsiveness to glucocorticoids in MDA-MB-231 cells (Ferrand et al. 2012), the two longer promoter constructs (− 1800/+ 21 and − 1191/+ 21) conferred reporter gene activity in response to dexamethasone in primary cFBs, IMR-90 cells, and human vein endothelial cells (HUVEC) (Figs. 3d, SI-c2, and SI-c3). In contrast, the more severely truncated promoter constructs (− 884/+ 21 and − 398/+ 21) lacking the GRE element, did not confer responsiveness to dexamethasone (Figs. 3d and SI-c2). Thus, the segment of the CCN5 promoter containing the described GRE is necessary for glucocorticoid induced activation of the CCN5-promoter also in these cells.

### CCN5 expression is induced by the β-adrenergic agonist isoproterenol in vitro and in vivo

**Fig. 4:**
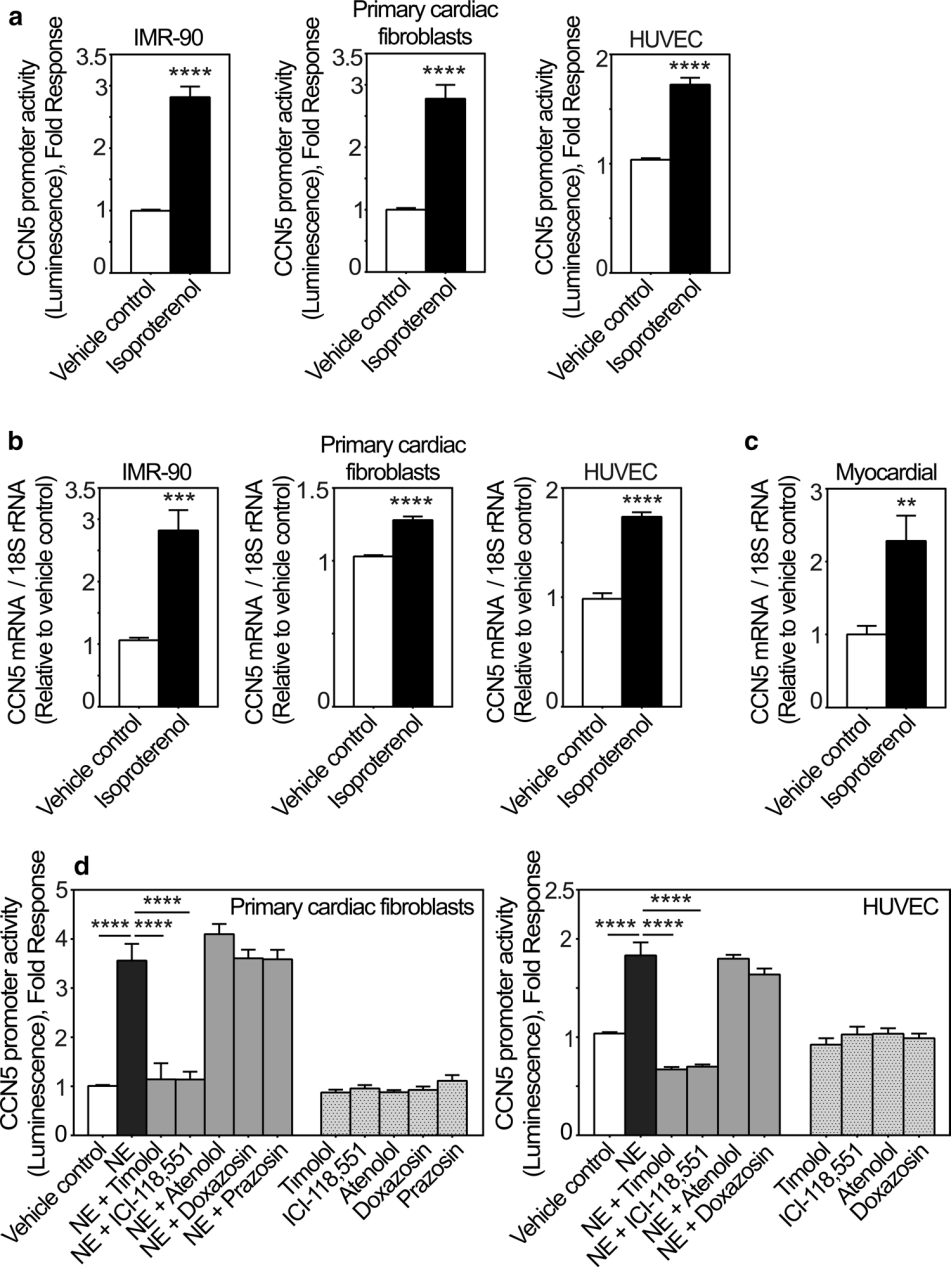
CCN5 transcriptional activity and mRNA levels are induced by catecholamines via β_2_-adrenergic receptors. **a** Histogram demonstrating transcriptional activity of the CCN5 promoter after stimulation of IMR-90 cells, primary cardiac fibroblasts and HUVEC cells in the absence or presence of isoproterenol. Following infection with recombinant adenovirus encoding luciferase under control of the CCN5 promoter fragment, IMR-90 cells, primary cardiac fibroblasts and HUVEC cells were stimulated with 200 nM isoproterenol or vehicle for 24 h and assayed for luciferase activity. The results were normalized to vehicle control and represent as the mean ± SEM (n ≥ 3 independent experiments each with three replicates per condition for IMR-90 and HUVEC cells and two replicates per condition for primary cardiac fibroblasts. **b** CCN5 mRNA levels in IMR-90 cells, primary cardiac fibroblasts and HUVEC cells stimulated in the absence or presence of isoproterenol. CCN5 mRNA levels were analyzed by real-time RT-PCR. Cells were seeded out and starved in 0.1% FBS for 24 h and subsequently, stimulated with 200 nM isoproterenol or vehicle for 72 h. The results were normalized to vehicle control and represent as the mean ± SEM (n = 3 independent experiments with two replicates per condition). **c** Myocardial CCN5 mRNA levels in mice subjected to continuous subcutaneous infusion of isoproterenol (50 mg/kg per day via micro-osmotic pumps) or vehicle for 14 days. Myocardial CCN5 mRNA levels were analyzed by real-time RT-PCR. The results were normalized to levels in vehicle control group and presented as the mean ± SEM (n = 5 isoproterenol-infused and n = 6 vehicle control mice). Statistical significance was calculated by unpaired Student’s two tailed t-test. **p < 0.01, ***p < 0.001 and ****p < 0.0001. Panel **d** shows histogram of the CCN5 promoter activity in primary cardiac fibroblasts and HUVEC cells transduced with recombinant adenovirus encoding luciferase under control of the CCN5 promoter and subsequently stimulated in the absence or presence of 20 µM norepinephrine (NE) alone, or in combination with either 100 nM timolol, 100 nM ICI-118,551, 1 µM atenolol, 10 nM doxazosin, 10 µM prazosin, or vehicle for 24 h. The results were normalized to vehicle control and presented as the mean ± SEM (n ≥ 3 independent experiments each with two replicates (primary cardiac fibroblasts) or three replicates (HUVEC cells) per condition). Statistical significance was calculated by one-way ANOVA with Šidák’s post hoc test. ****p < 0.0001 versus NE group

During the proliferation and maturation phases of wound healing following MI, when CCN5 is upregulated in the infarct region, neurohormonal activity is also often increased. Hence we investigated the effect of adrenergic stimulation on CCN5 expression in fibroblasts and endothelial cells, as they were identified to be major producers of CCN5 in the infarcted tissue (Fig. 2). As shown in Fig. 4a, isoproterenol, a non-selective β-adrenergic receptor agonist, stimulated CCN5 promoter activity in primary cFBs, IMR-90 cells, and HUVEC cells. Isoproterenol also increased CCN5 mRNA levels in primary cFBs, IMR-90 cells, and HUVEC cells following stimulation (Fig. 4b), and similarly in the hearts of mice subjected to isoproterenol infusion in vivo (Fig. 4c), the latter suggesting that β-adrenergic stimulation is also participating in regulation of CCN5 expression in vivo.

### The stimulatory effect of adrenergic agonists on the CCN5 promoter activity is dependent on β_2_-adrenergic receptors

In order to decipher what β-adrenergic receptor subtype is responsible for the responsiveness of the CCN5 promoter to β-adrenergic stimulation, cFBs, HUVEC cells, and IMR-90 cells were stimulated with norepinephrine in the presence or absence of the following adrenergic receptor-antagonists; timolol (a non-selective β-AR antagonist); ICI-118,551 (a selective β_2_-AR antagonist); atenolol (a selective β_1_-AR antagonist); doxazosin and prazosin (selective α_1_-AR antagonists), or incubated in the presence of the receptor antagonists alone. As shown in Figs. 4d and SI-d, norepinephrine-stimulated activation of the CCN5 promoter was not sensitive to atenolol, doxazosin or prazosin. However, the norepinephrine-stimulated activation of the CCN5-promoter was sensitive to both timolol and ICI-118,551, thus validating that the stimulatory effect of adrenergic agonists is dependent on β-receptors, and specifically β_2_-receptors.

### The second messenger cAMP stimulates CCN5 gene expression in fibroblasts

**Fig. 5:**
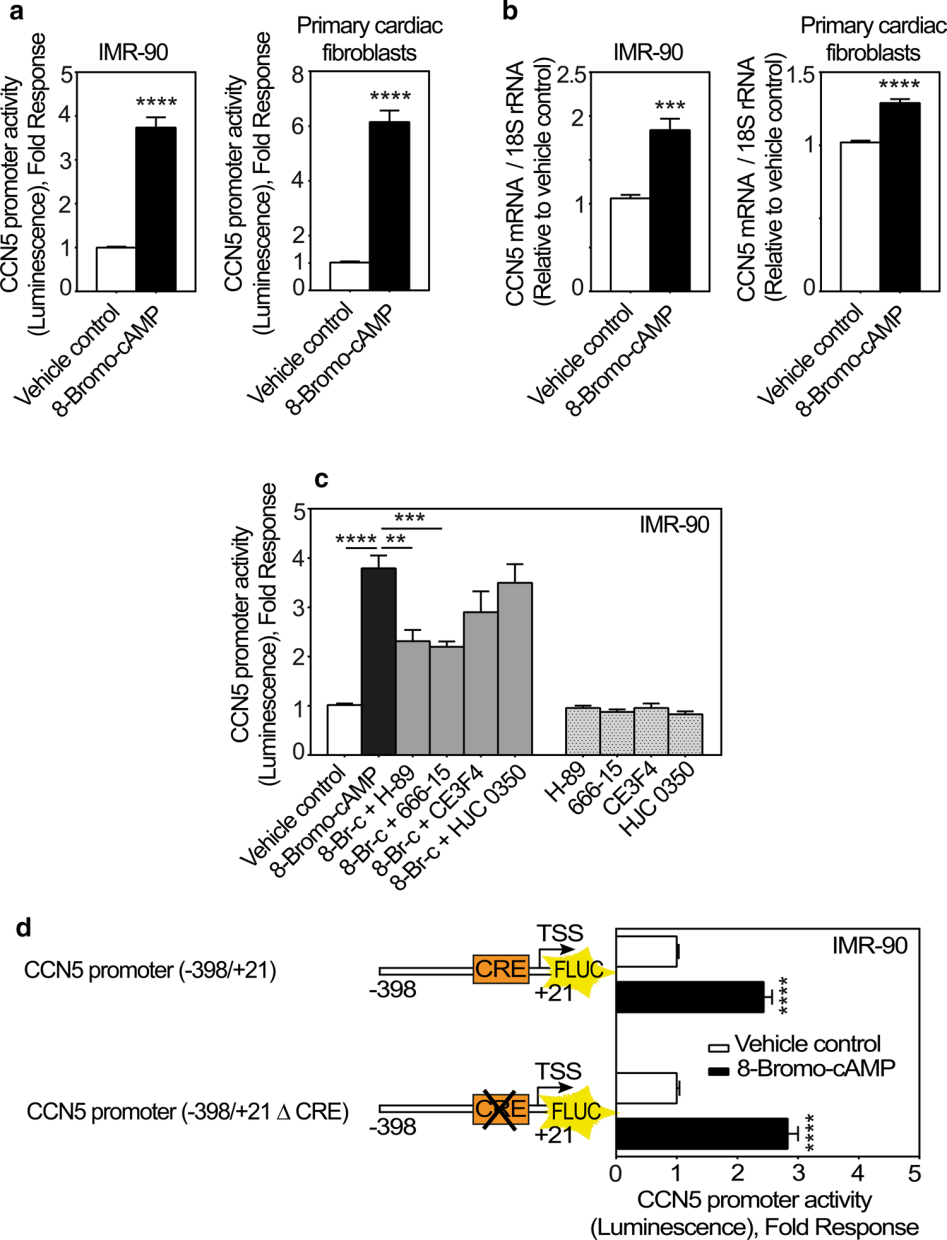
CCN5 promoter activity is enhanced by 8-Bromo-cAMP. **a** Histogram demonstrating transcriptional activity of the CCN5 promoter following stimulation of IMR-90 cells or primary cardiac fibroblasts with 8-Bromo-cAMP. Briefly, the cells were infected with recombinant adenovirus encoding luciferase under control of the CCN5 promoter (− 1800/+ 21). After transduction, cells were stimulated with 1 mM 8-Bromo-cAMP or vehicle for 24 h and subsequently assayed for luciferase activity. The results were normalized to vehicle control and presented as the mean ± SEM (n ≥ 3 independent experiments with each three replicates (IMR-90 cells) or two replicates (primary cardiac fibroblasts) per condition). **b** Histogram demonstrating CCN5 mRNA levels in IMR-90 cells and primary cardiac fibroblasts following stimulation with 8-Bromo-cAMP. Cells were seeded out and starved in 0.1% FBS for 24 h and then stimulated with 1 mM 8-Bromo-cAMP or vehicle for 72 h. mRNA levels were assayed by real-time RT-PCR. The results were normalized to vehicle control and presented as the mean ± SEM (n = 3 independent experiments each with two replicates per condition). **c** Histogram demonstrating transcriptional activity of the CCN5 promoter following stimulation of IMR-90 cells with 1 mM 8-Bromo-cAMP (8-Br-c) alone or in combination with 100 nM H-89, 100 nM 666-15, 100 nM CE3F4, 100 nM HJC 0350, or vehicle for 24 h. The results were normalized to vehicle control and represent the mean ± SEM (n = 3 independent experiments each with three replicates per condition). Statistical significance was calculated by one-way ANOVA with Šidák’s post hoc test. **p < 0.01, ***p < 0.001 and ****p < 0.0001 versus 8-Bromo-cAMP group. **d** Schematic and histogram demonstrating responsiveness of a CCN5 promoter fragment to 8-Bromo-cAMP in the absence or presence of CRE-element. Briefly, IMR-90 cells were infected with recombinant adenovirus encoding luciferase under control of − 398/+ 21 CCN5 promoter fragment with or without deletion of the CRE element. Following viral transduction, the cells were stimulated in the absence or presence of 8-Bromo-cAMP (1 mM) for 24 h before recording of luciferase activities. The results were normalized to vehicle control and represent the mean ± SEM (n = 3 independent experiments each with three replicates per condition). Statistical significance was calculated by unpaired Student’s two tailed t-test. ****p < 0.0001

The major intracellular mediator generated by β_2_-adrenergic receptor activation is cAMP. To investigate to what extent cAMP may convey stimulation of CCN5-promoter activity; we stimulated cFBs and IMR-90 cells with 8-bromo-cAMP (a brominated derivative of cAMP with cell membrane permeability and resistance to phosphodiesterases). Indeed, as shown in Fig. 5a, 8-bromo-cAMP induced CCN5 promoter activity in both IMR-90 cells and cFBs. In congruence with these findings, CCN5 mRNA levels were also upregulated following stimulation with 8-Bromo-cAMP (Fig. 5b) in cFBs and IMR-90 cells. To investigate whether the responsiveness of the CCN5 promoter to 8-Bromo-cAMP is mediated by protein kinase A (PKA) or exchange proteins directly activated by cAMP (EPACs), IMR-90 cells were stimulated with 8-Bromo-cAMP in the presence or absence of H-89 (an inhibitor of protein kinase A (PKA)); 666-15 (a selective inhibitor of CREB); CE3F4 (a selective inhibitor of EPAC1); HJC 0350 (a selective inhibitor of EPAC2). As shown in Fig. 5c, 8-Bromo-cAMP-stimulated activation of the CCN5 promoter was sensitive to neither CE3F4 nor HJC 0350. However, the 8-Bromo-cAMP-stimulated activation of the CCN5-promoter was sensitive to both H-89 and 666-15. Thus, these results led us to hypothesize that cAMP-induced activation of the CCN5 promoter activity is conferred by the canonical cAMP response element (CRE) predicted to be present in the CCN5 promoter (Fritah et al. 2006). However, deletion of the CRE element from the CCN5 promoter did not abrogate 8-Bromo-cAMP-stimulated CCN5 transcriptional activity, indicating that this activity was mediated by a mechanism not dependent on the CRE element (Fig. 5d).

### CCN5 protein levels are increased following β_2_-adrenergic stimulation in IMR-90 cells

**Fig. 6:**
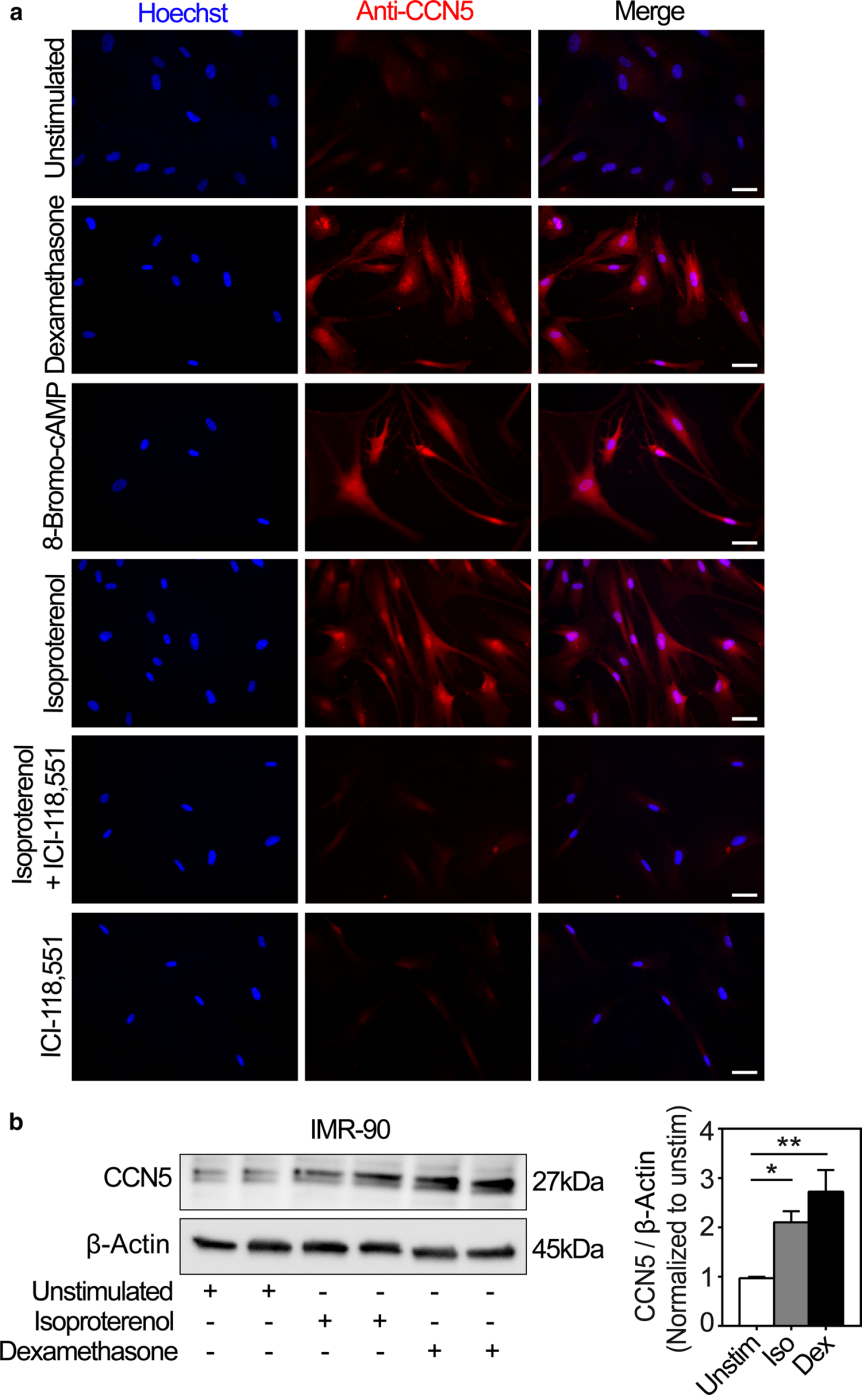
Immunocytochemical and Western blot analyses of CCN5 in IMR-90 cells subjected to diverse exposures. **a** Panels are photomicrographs of IMR-90 cells subjected to immunofluorescence analysis of CCN5 immunoreactivity after stimulation of the cells in the absence or presence of 100 nM glucocorticoid (dexamethasone), 1 mM 8-Bromo-cAMP, 200 nM isoproterenol, and 100 nM ICI-118,551 for 72 h. Nuclear DNA was stained with Hoechst 33258 (blue). Images are representative three independent experiments with 2 replicates per condition. More than 360 cells were analyzed per experimental condition. Scale bar is 50 µM. **b** Total cell lysates from IMR-90 cells stimulated in the absence or presence of isoproterenol (Iso; 200 nM) or dexamethasone (Dex; 100 nM) for 72 h were separated by SDS gel electrophoresis, transferred to PVDF membrane, and immunoblotted with antibodies against CCN5 and β-Actin. The photomicrograph demonstrates the immunoreactive bands and is representative of three independent experiments with 2 replicates per condition. The histogram shows densitometric analysis of CCN5 levels relative to β-actin (loading control) of the three independent experiments presented as mean ± SEM. Statistical significance was calculated by one-way ANOVA with Dunnett’s post hoc test. *p < 0.05 and **p < 0.01 versus unstimulated (Unstim)

To investigate whether the β_2_-adrenergic stimulation of CCN5 promoter activity and CCN5 gene expression also translated into increased protein expression, IMR-90 cells were stimulated in the presence or absence of isoproterenol with or without ICI-118,551 and analyzed for increase of CCN5 immunoreactivity by immunocytochemistry. As shown in Fig. 6a, stimulation of IMR-90 cells in the presence of dexamethasone, isoproterenol or 8-Bromo-cAMP all substantially enhanced CCN5 immunoreactivity. Furthermore, stimulation of the cells with isoproterenol in the presence of ICI-118,551 essentially abolished CCN5 immunostaining, corroborating the finding of β_2_-adrenergic receptor-mediated activation of CCN5 transcription in IMR-90 cells. Consistent with the immunocytochemistry studies, Western blot analysis of IMR-90 cells stimulated with isoproterenol (200 nM) or dexamethasone (100 nM) also demonstrated increased CCN5 levels (Fig. 6b).

### TNF-α inhibits expression of CCN5 in fibroblasts and endothelial cells

**Fig. 7:**
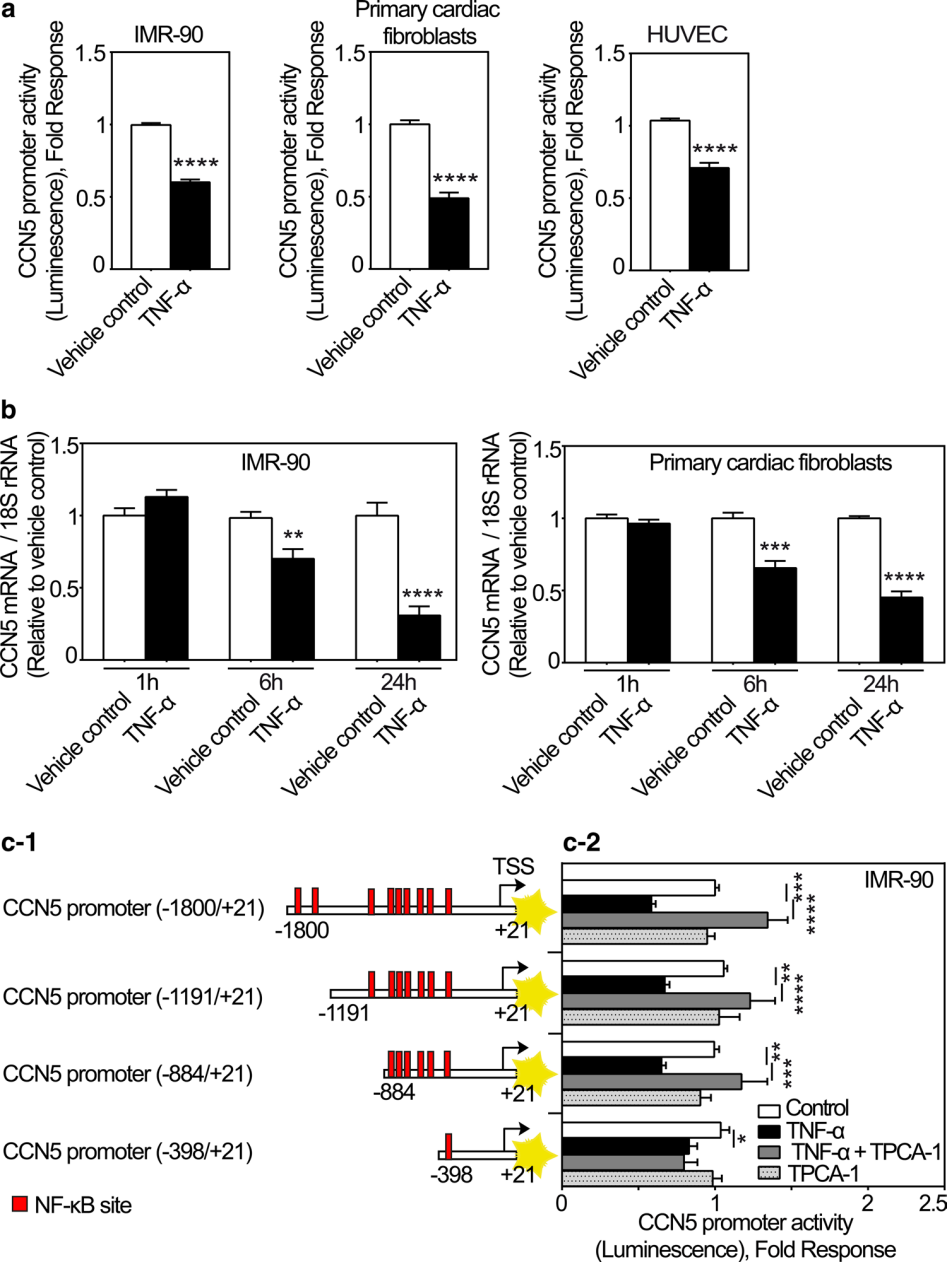
CCN5 transcription is inhibited by TNF-α. **a** Histogram demonstrating transcriptional activity of the CCN5 promoter after stimulation of IMR-90 cells, primary cardiac fibroblasts and HUVEC cells in the absence or presence of TNF-α. Following infection with recombinant adenovirus encoding luciferase under control of the CCN5 promoter fragment, IMR-90 cells, primary cardiac fibroblasts and HUVEC cells were stimulated with TNF-α (100 ng/ml) or vehicle for 24 h and assayed for luciferase activity. The results were normalized to vehicle control and represent as the mean ± SEM (n ≥ 3 independent experiments each with three replicates (IMR-90 and HUVEC cells) or two replicates (primary cardiac fibroblasts) per condition). **b** Histogram demonstrating CCN5 mRNA levels in IMR-90 cells and primary cardiac fibroblasts following stimulation in the absence or presence of TNF-α. The cells were seeded out and starved in 0.1% FBS for 24 h and subsequently, stimulated with 100 ng/ml TNF-α or vehicle for 1, 6 and 24 h in both IMR-90 cells and primary cardiac fibroblasts. CCN5 mRNA levels were analyzed by real-time RT-PCR. The results were normalized to vehicle control and presented as the mean ± SEM (n = 3 independent experiments each with two replicates per condition). Statistical significance was calculated by unpaired Student’s two tailed t-test. **p < 0.01, ***p < 0.001 and ****p < 0.0001. **c-1** Schematic demonstrating various CCN5 promoter fragments containing several putative NF-κB sites. **c-2** After infection of IMR-90 cells with recombinant adenovirus encoding firefly luciferase under control of the CCN5 promoter fragment with various deletions, the cells were stimulated with 100 ng/ml TNF-α alone or in combination with 10 µM TPCA-1 or vehicle for 24 h and assayed for luciferase activity. The results were normalized to vehicle control and presented as the mean ± SEM (n ≥ 3 independent experiments each with three replicates per condition). Statistical significance was calculated by one-way ANOVA with Šidák’s post hoc test. *p < 0.05, **p < 0.01, ***p < 0.001 and ****p < 0.0001 versus TNF-α group

Although catecholamines were shown to increase the expression of CCN5 via β-adrenergic receptor activation, no increase of cardiac mRNA levels of CCN5 was observed in the early inflammatory phase following MI. During this period, the presence of inflammatory cytokines is known to be high (Nian et al. 2004). Hence, we hypothesized that expression of CCN5 might be repressed by an inflammatory cytokine during first days following MI. TNF-α is a proinflammatory cytokine elevated in the early phase of wound healing after myocardial infarction as well as in progression of ischemic heart failure and is associated with advancing fibrosis (Herskowitz et al. 1995; Nian et al. 2004; Kleinbongard et al. 2010). As shown in Fig. 7a TNF-α inhibited the CCN5 promoter activity in cFBs, IMR-90 cells, and HUVEC cells. Consistently, TNF-α also swiftly reduced CCN5 mRNA levels in both cFBs and IMR-90 cells (Fig. 7b). There are several predicted consensus NF-κB suppressor elements along the promoter of CCN5 (Fig. 7c-1). To investigate to what extent the repressive effect of TNF-α on CCN5 promoter activity was dependent on NF-κB signaling, IMR-90 cells were transduced with recombinant adenovirus containing varying lengths of the CCN5 promoter (− 1800/+ 21, − 1191/+ 21, − 884/+ 21 and − 398/+ 21) set to control the firefly luciferase (FLUC) reporter gene (Fig. 7c-2), and subsequently stimulated with TNF-α in the presence or absence of TPCA-1 (a selective inhibitor of IKK-2 of the NF-κB pathway) or incubated with TPCA-1 alone. All promoter constructs (− 1800/+ 21, − 1191/+ 21, − 884/+ 21 and − 398/+ 21) conferred inhibition of reporter gene activity in response to TNF-α. In addition, TPCA-1 abolished the inhibitory effect of TNF-α at the CCN5 promoter variants except for the most severely truncated promoter construct (− 398/+ 21) having only one NF-κB site (Fig. 7c-2). Thus, the repression of transcription from the CCN5 promoter appears to be mediated by IKK-2-dependent activity at the distal NF-κB sites present from nucleotides − 1800 to − 398 of the CCN5 promoter.

## Discussion

The present study reports data from investigations on transcriptional regulation and analysis of cellular distribution of CCN5 in the heart after myocardial infarction. The study discloses the novel findings that CCN5 is transcriptionally induced by catecholamines acting through β_2_-adrenergic receptors on endothelial cells and cardiac fibroblasts and repressed by TNF-α through NF-κB suppressor elements, i.e. factors that are elevated following myocardial infarction and during ischemic heart failure. Further, high mRNA and protein levels of CCN5 were found in the granulation tissue and differentiating scar tissue after myocardial infarction. CCN5 immunoreactivity was predominantly confined to endothelial cells, fibroblast-like cells, and mononuclear leukocytes, similar to what has previously been reported for CCN2. However, induction of CCN5 was delayed compared with that of CCN2. Rising levels of CCN5 were first observed in the proliferative and maturation phases of wound healing suggesting disparate roles of CCN2 and CCN5.

CCN2 is considered an important mediator of myocardial fibrosis elevated in myocardial tissue in animal models as well as in patients with heart failure (Chen et al. 2000; Ahmed et al. 2004). As a diverging member of the CCN family, CCN5 is a particularly interesting molecule due to its reported ability to counteract the pro-fibrotic activity of CCN2 (Yoon et al. 2010). Yoon et al., showed that cardiac-restricted overexpression of CCN5 in genetically engineered mice reduced cardiac hypertrophy and fibrosis following pressure overload. The mechanisms of the opposing actions of CCN5 versus CCN2 is yet unknown, but could be related to the lack of the carboxyl-terminal cystine-knot domain present in 4-domain CCN proteins. Indeed, CCN5 with the cystine knot domain of CCN2 appended to its C-terminal end was shown to attain properties similar to CCN2 in human skin fibroblasts (Xu et al. 2015). The regulation of CCN5 has also been found to differ from the regulation of CCN1, CCN2 and CCN3 in mouse embryos (Myers et al. 2012), indicating that CCN5 is subject to different regulatory mechanisms than the other CCN family members during embryonic development. The delayed increase of CCN5 mRNA levels relative to that of CCN2 mRNA levels in the infarcted region indicates that CCN2 and CCN5 may exert different actions during wound healing and tissue repair. Similar to CCN2, myocardial CCN1 and CCN4 also respond as immediate early genes and reach peak levels of expression during the inflammatory phase after onset of myocardial ischemia (Hilfiker-Kleiner et al. 2004; Colston et al. 2007; Jun et al. 2015).

In order to explore the factors that control CCN5 activity, we investigated transcriptional regulation from the CCN5 promoter. CCN5 has been previously reported to be transcriptionally induced by oestrogens and glucocorticoids in breast cancer cells (Banerjee et al. 2003; Fritah et al. 2006; Ferrand et al. 2012). The glucocorticoid response to CCN5 in MDA-MB-231 breast cancer cells was mapped to a glucocorticoid response element (GRE) in the promoter region of CCN5 (Ferrand et al. 2012). In this study, we also find that dexamethasone activates transcription from the CCN5 promoter in fibroblasts and that this response is lost in a truncated promoter lacking the GRE element. Similarly, it has previously been reported that an estrogen receptor element of the CCN5 promoter confers CCN5 transcription in response to estrogens (Fritah et al. 2006). However, in the same paper Fritah et al. (2006) also disclosed that the promoter region of CCN5 also contained consensus sequences for AP-1 enhancer elements, SP-1 elements, and CRE elements. Thus, neurohormonal activation subsequent to myocardial infarction and impairment of cardiac function may conceivably cause activation of transcription from the CCN5 promoter.

In this respect, elevation of myocardial CCN5 mRNA levels following chronic infusion of isoproterenol in mice is congruent with the finding of β_2_-adrenergic receptor-mediated activation of transcription from the CCN5 promoter in cardiac fibroblasts and endothelial cells as reported in this study. Indeed, cardiac fibroblasts and endothelial cells have previously been found to predominantly express the β_2_-adrenergic receptor subtype (Lau et al. 1980; Broeders et al. 2000; Kim et al. 2002). Although the mechanisms of CCN5-mediated inhibition of fibrosis have not yet been resolved, the finding that CCN5 is induced by β_2_-adrenergic receptor agonists is intriguing in view of a report showing that β_2_-adrenergic receptor stimulation may promote collagen degradation and prevent fibrosis (Aranguiz-Urroz et al. 2011).

Cyclic-AMP, the major intracellular transmitter of β-adrenergic receptor activation, would be the principal candidate to confer catecholamine-stimulated transcription from the CCN5 promoter. Indeed, the cell permeable cyclic-AMP analog 8-bromo-cyclic-AMP, elicited robust transcription from the CCN5 promoter. Yet, although our data indicate that catecholamine-stimulated transcription from the CCN5 promoter is mediated via protein kinase A, the downstream mechanism remains to be resolved. The fact that elimination of the putative CRE element by in situ mutagenesis did not abrogate 8-bromo-cyclic-AMP-mediated transcription from the CCN5 promoter suggests that cyclic-AMP-induced transcription of CCN5 may be conferred by a PKA-dependent mechanism that does not involve CREB and the CRE element. In this respect, it should be emphasized that both the PKA inhibitor (H-89) and the highly selective CREB inhibitor (666-15) only partially inhibited cyclic-AMP-stimulated transcription from the CCN5 promoter. Thus, cyclic-AMP/PKA may cross-talk with other signaling pathways also involved in regulation of transcription from the CCN5 promoter. Indeed, PKA has been reported to catalyze phosphorylation of GSK-3β leading to TCF/LEF-mediated enhancement of transcription from the CCN5 promoter (McManus et al. 2005; Suzuki et al. 2008). Hence, non-CREB/CRE-dependent mechanisms for cyclic-AMP-mediated transcription from the CCN5 promoter appear to exist. Consistent with a cross-talk mechanism was also the observation that cyclic-AMP-induced elevation of CCN5 mRNA levels appeared to be a relatively slow response indicating that induction of CCN5 in this case may be due to a secondary mechanism. A plausible explanation why cyclic-AMP-stimulated transcription of CCN5 could not be conferred by the CRE-element in the CCN5 promoter is still obscure. Conceivably, the context surrounding the CRE element may not favor stimulation of transcription from this element.

The mechanisms of the delayed induction of CCN5 relative to CCN2 following myocardial infarction has not yet been resolved. However, the finding in this study that TNF-α represses transcription from the CCN5 promoter via NF-κB-responsive elements may provide a plausible explanation for the delayed induction of CCN5 following myocardial infarction as myocardial TNF-α reaches peak levels within 24 h after myocardial infarction (Herskowitz et al. 1995; Nian et al. 2004).

The finding that the delayed increase of mRNA and protein levels of CCN5 was primarily observed in the region subjected to ischemic damage, prompted us to investigate the cellular distribution of CCN5 in this region. In normal hearts, immunoreactive CCN5 could primarily be discerned in endothelial cells and smooth muscle cells of muscular arteries. In the differentiating scar tissue, immunoreactive CCN5 was found in endothelial cells and fibroblast-like cells as well as in macrophages. This cellular distribution is very similar to that previously observed for CCN2 and other 4-domain CCN proteins during formation of granulation tissue following myocardial necrosis (Ahmed et al. 2004; Colston et al. 2007; Jun et al. 2015) and may point to a role of CCN5 in inhibiting the other 4-domain CCN proteins. A former report showed that CCN5 may inhibit transdifferentiation of cardiac fibroblasts and endothelial cells to myofibroblasts in vitro (Jeong et al. 2016). The same report also provided evidence that CCN5 delivered to the heart by a recombinant adeno-associated virus vector caused apoptosis of activated myofibroblasts in the heart subjected to transverse aortic constriction and attenuated myocardial fibrosis. Endothelial cells are also critically involved in cardiac remodeling after myocardial infarction by actively secreting proteins such as extracellular matrix (ECM) proteins and pro-fibrotic factors (Segers et al. 2018) to regulate functions of neighboring cells. Interestingly, previous findings indicate that CCN5 inhibits endothelial-mesenchymal transition (EndMT) both in vivo and in vitro by downregulating fibroblast-associated genes and upregulating endothelial-associated markers, thus preventing transition of endothelial cells to myofibroblasts (Jeong et al. 2016). Scar-tissue macrophages may also be involved in the pathophysiology of myocardial remodeling, in particular if tissue injury persists, by releasing proinflammatory cytokines, growth factors, and pro-fibrotic factors (Nikolic-Paterson et al. 2014). Of note, CCN2 has been reported to promote inflammation and infiltration of macrophages in vitro and in vivo under inflammatory conditions (Charrier et al. 2014). Considering the various reports on converse actions of CCN5 and CCN2, it is conceivable that CCN5 secreted from fibroblasts, endothelial cells and macrophages may work in an autocrine/paracrine manner to modify the actions of CCN2 (and other four-domain CCN proteins) on fibroblasts and thereby inhibit fibrosis and contribute to resolution of the wound healing process. Based on evidence that CCN5 inhibits fibrosis (Yoon et al. 2010; Jeong et al. 2016; Huang et al. 2020), the delayed increase of CCN5 levels may function to curb excessive scar formation.

**Fig. 8:**
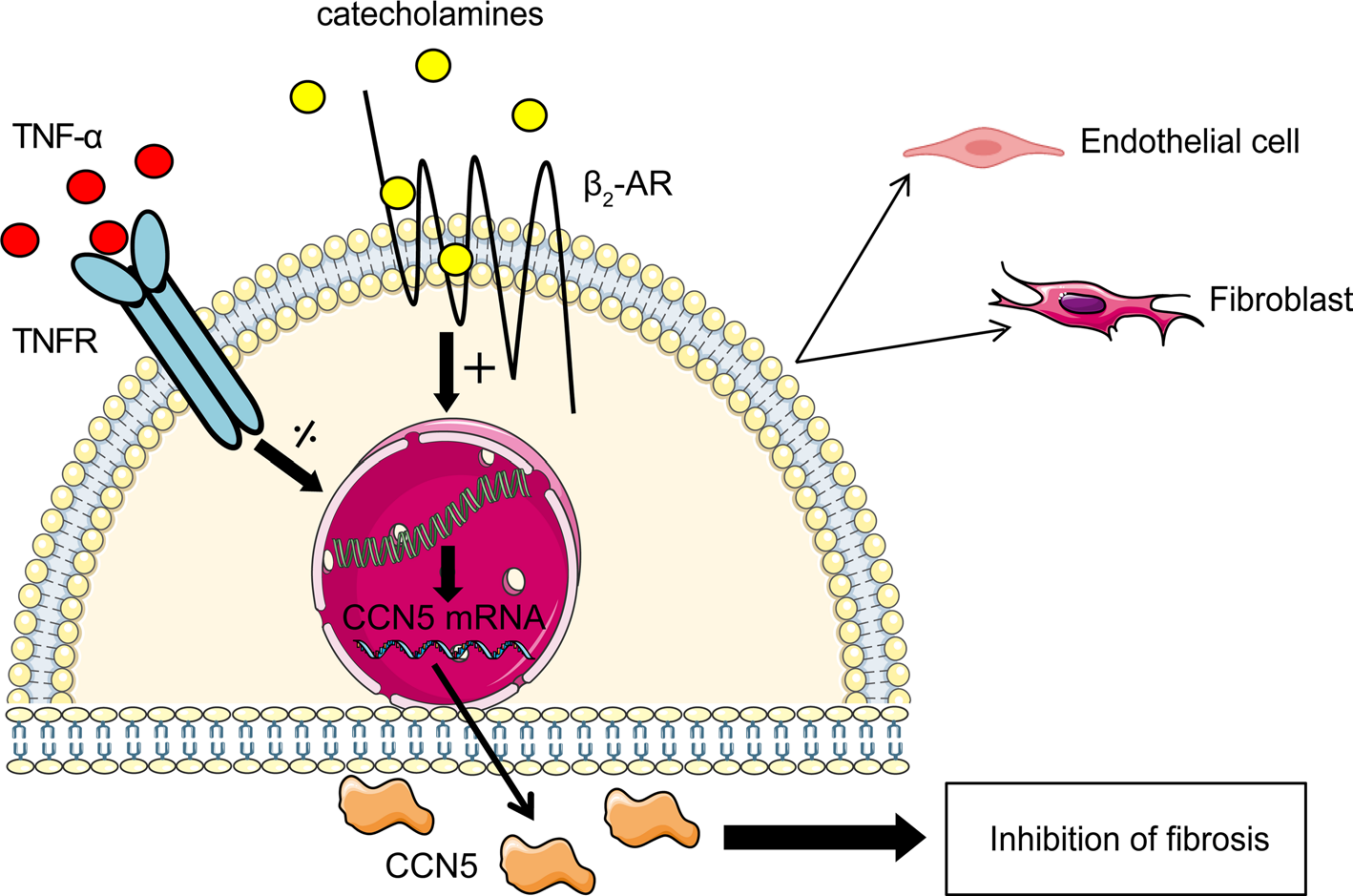
Schematic model of transcriptional regulation of CCN5 in endothelial cells and fibroblasts of the heart. CCN5 promoter activities are conversely regulated by catecholamines and TNF-α in endothelial cells and fibroblasts

In conclusion, this study discloses the novel findings that transcription from the CCN5 promoter is stimulated by catecholamines via the β_2_-adrenergic receptors on cardiac fibroblasts and endothelial cells and repressed by TNF-α through NF-κB suppressor elements in the promoter region of CCN5 (Fig. 8). The early inflammatory phase following myocardial infarction with soaring levels of TNF-α may provide basis for the delayed increase of CCN5 relative to CCN2.

## Supplementary Information

Below is the link to the electronic supplementary material.

Supplementary file1 (PDF 568 kb)

## Data Availability

The data underlying this article are available from the corresponding author on reasonable request.

## Abbreviations

AR: Adrenergic receptor
CCN: Cellular communication network factor
cFB: Cardiac fibroblast
ChIP: Chromatin immunoprecipitation
CRE: cAMP response element
CT: C-terminal
CTGF: Connective tissue growth factor
DMEM: Dulbecco’s modified Eagle’s medium
EBM: Endothelial cell basal medium
ECM: Extracellular matrix
EndMT: Endothelial-mesenchymal transition
EPACs: Exchange proteins activated by cAMP
FBS: Fetal bovine serum
FLUC: Firefly luciferase
GRE: Glucocorticoid response element
HUVEC: Human umbilical vein endothelial cells
IF: Immunofluorescence
IGFBP: Insulin-like growth factor-binding protein
IHC: Immunohistochemistry
LAD: Left anterior descending
MI: Myocardial infarction
NE: Norepinephrine
PKA: Protein kinase A
q-RT-PCR: Quantitative real-time PCR
α-SMA: α-smooth muscle actin
TGF-β: Transforming growth factor-β
TNF-α: Tumor necrosis factor-α
TNFR: Tumor necrosis factor receptor
TSS: Transcription start site
UCSC: University of California, Santa Cruz
VWC: von Willebrand factor type C
WISP-2: WNT1 inducible signaling pathway protein-2
