# Supplementary material for: Tissue distribution and transcriptional regulation of CCN5 in the heart after myocardial infarction
Source: J Cell Commun Signal. 2021 Dec 1;16(3):377–95. doi: 10.1007/s12079-021-00659-7 (PMC9411331; doi:10.1007/s12079-021-00659-7)
Supplement: Supplementary file 1 — Supplementary file1 (PDF 568 kb) [file 12079_2021_659_MOESM1_ESM.pdf]

Tissue distribution and transcriptional regulation of CCN5 in the heart after myocardial infarction

Journal of Cell Communication and Signaling

Sima Zolfaghari[1, 2], Ole Jørgen Kaasbøll[1], M. Shakil Ahmed[1], Fabian A. Line[1, 2], Else Marie V. Hagelin[1], Vivi T. Monsen[1, 2], and Håvard Attramadal[1, 2]

Affiliations:

[1]Institute for Surgical Research, Oslo University Hospital, Oslo, Norway

[2]Institute of Clinical Medicine, University of Oslo, Norway

Corresponding author:

Håvard Attramadal, M.D., Ph.D.
E-mail: havard.attramadal@medisin.uio.no
ORCID: 0000-0001-9506-0687
Institute for Surgical Research, Rm. A3.1056
Oslo University Hospital
Sognsvannsveien 20
P.O. Box 4950 Nydalen
0424 OSLO
Norway
Phone: +47-23073532

Supplementary Information (SI)

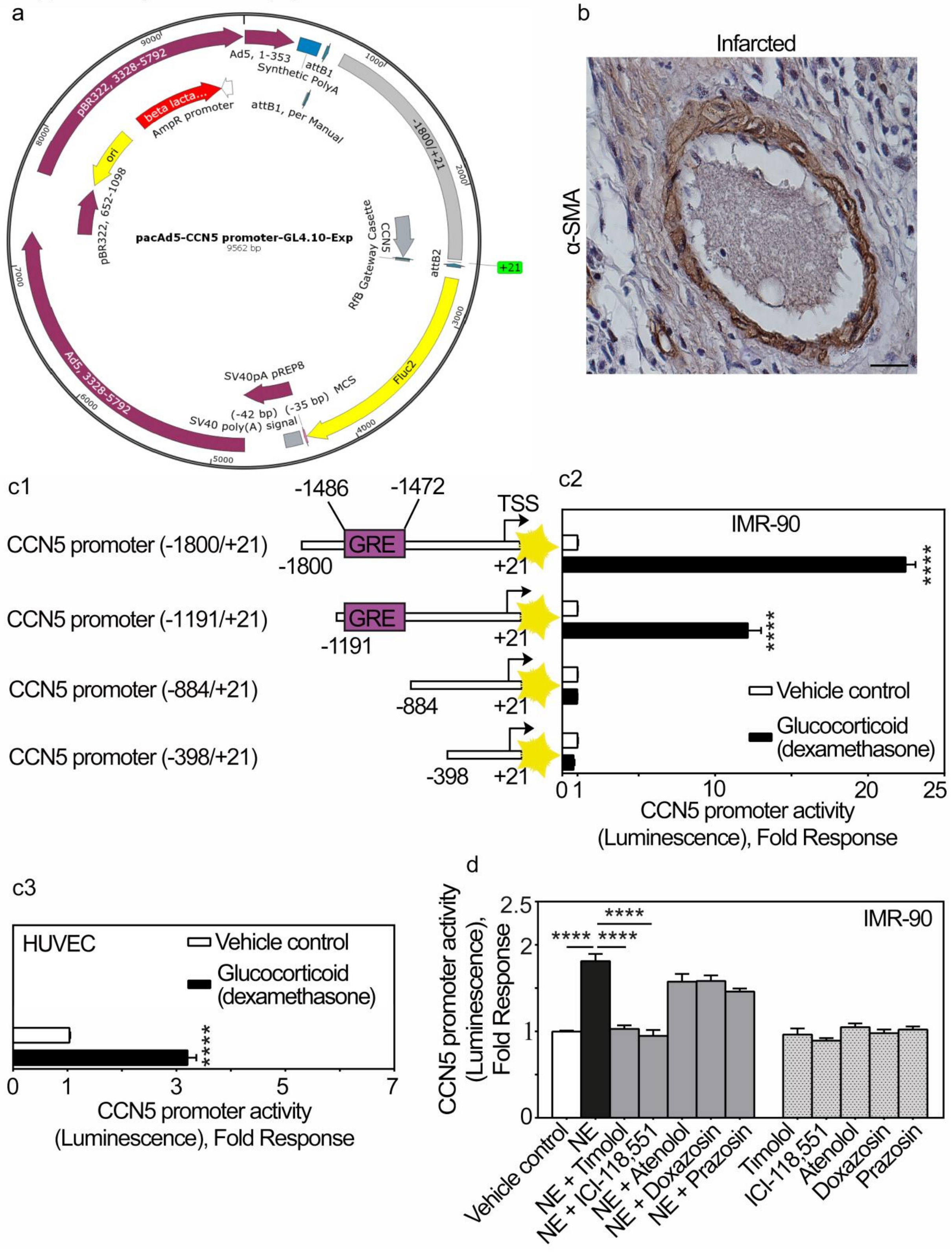

Supplementary Information (SI)

(a). Schematic of generated CCN5 promoter adenoviral vector. Schematic illustrates pacAd5-pGL4.10-Exp adenoviral shuttle vector containing the coding sequence of firefly luciferase flanked at its 5'-end by the human CCN5 promoter and transcriptional start site (-1800/+21). The recombinant shuttle vector was generated by standard Gateway™ ligation-independent cloning with attB Gateway™ recombination sequences flanking the cassette to be inserted in the pacAd5 shuttle vector. The schematic map was created with the SnapGene program 5.3.2

(b). Immunohistochemical analysis of α-SMA immunoreactivity in myocardial tissue. Panel (**b**) is photomicrograph of immunohistochemistry of α-smooth muscle actin (α-SMA) immunoreactivity in myocardial tissue section from the infarct region of mice 4 weeks after permanent ligation of the left coronary artery and induction of myocardial infarction. α-SMA immunoreactivity was identified in smooth muscle cells of the muscular layer of a vessel. Photomicrograph is representative immunostaining of myocardial sections from 2 mice subjected to myocardial infarction (three myocardial sections were subjected to the indicated immunostaining). Scale bar is 20 µM

(c1, 2, and 3). Schematic of CCN5 promoter-reporter constructs and deletion analysis of the CCN5 promoter and response to glucocorticoids. (**c1**) Schematic demonstrating various deletions of the -1800/+21 promoter construct with indications of the previously identified glucocorticoid response (enhancer) element (GRE). After infection of IMR-90 cells (**c2**) with recombinant adenovirus encoding luciferase under control of the CCN5 promoter fragment with various deletions, the cells were stimulated with 100 nM dexamethasone or vehicle for 24 hours and assayed for luciferase activity. Panel (**c3**) represents HUVEC cells infected with recombinant adenovirus encoding luciferase under control of the -1800/+21 promoter fragment and stimulated with 100 nM dexamethasone or vehicle for 24 hours and assayed for luciferase activity. The results represent the mean ± SEM ($n \geq 3$ independent experiments

with three replicates per condition) of luciferase activity in IMR-90 cells and HUVEC cells stimulated in the absence or presence of dexamethasone. Statistical significance was assessed by unpaired Student's two tailed t-test. ****P < 0.0001

(d). Norepinephrine-stimulated CCN5 promoter activity in IMR-90 cells is sensitive to inhibition by β_2-adrenoceptor antagonists. Panel (**d**) shows histogram of the CCN5 promoter activity in IMR-90 cells transduced with recombinant adenovirus encoding luciferase under control of the CCN5 promoter and subsequently stimulated in the absence or presence of 20 µM norepinephrine (NE) alone, or in combination with either 100 nM timolol, 100 nM ICI-118,551, 1 µM atenolol, 10 nM doxazosin, 10 µM prazosin, or vehicle for 24 hours. The results were normalized to vehicle control and are presented as the mean ± SEM (n ≥ 3 independent experiments with three replicates per condition). Statistical significance was calculated by one-way ANOVA with Šidák's post hoc test. ****p < 0.0001 vs. NE group
